# Healthy Planet, Healthy Youth: A Food Systems Education and Promotion Intervention to Improve Adolescent Diet Quality and Reduce Food Waste

**DOI:** 10.3390/nu11081869

**Published:** 2019-08-11

**Authors:** Melissa Pflugh Prescott, Xanna Burg, Jessica Jarick Metcalfe, Alexander E. Lipka, Cameron Herritt, Leslie Cunningham-Sabo

**Affiliations:** 1 Department of Food Science and Human Nutrition, University of Illinois at Urbana-Champaign, Champaign, IL 61820, USA; 2 Department of Crop Sciences, University of Illinois at Urbana-Champaign, Champaign, IL 61820, USA; 3 Department of Food Science and Human Nutrition, Colorado State University, Fort Collins, CO 80523, USA

**Keywords:** food systems, school nutrition, food waste, adolescents, implementation science

## Abstract

Emerging evidence suggests a link between young people’s interest in alternative food production practices and dietary quality. The primary purpose of this study was to examine the impact of a student-driven sustainable food systems education and promotion intervention on adolescent school lunch selection, consumption, and waste behaviors. Sixth grade science teachers at two middle schools (*n* = 268 students) implemented a standards-based curriculum on sustainable food systems, addressing the environmental impacts of food choices and food waste. The cumulating curriculum activity required the 6th grade students to share their food systems knowledge with their 7th and 8th grade counterparts (*n* = 426) through a cafeteria promotional campaign to discourage food waste. School-wide monthly plate waste assessments were used to evaluate changes in vegetable consumption and overall plate waste using a previously validated digital photography method. At baseline, the intervention students consumed significantly less vegetables relative to the control group (47.1% and 71.8% of vegetables selected, respectively (*p* = 0.006). This disparity was eliminated after the intervention with the intervention group consuming 69.4% and the control consuming 68.1% of selected vegetables (*p* = 0.848). At five months follow up, the intervention group wasted significantly less salad bar vegetables compared to the control group (24.2 g and 50.1 g respectively (*p* = 0.029). These findings suggest that food systems education can be used to promote improved dietary behaviors among adolescent youth.

## 1. Introduction

School meal programs combat childhood hunger and inadequate nutrition by providing children with the nutrients needed for physical and educational development. These programs also present an important opportunity to simultaneously address child diet quality and food waste. About 95% of U.S. children aged 9–18 do not meet the federal dietary recommendations for vegetable intake [1,2,3], and childhood obesity continues to be a major public health problem [4]. U.S. Students who participate in both the National School Lunch Program (NSLP) and School Breakfast Program consume up to 47% of their daily energy intake from school meals [5], and school nutrition programs reduce household income disparities in adolescent fruit and vegetable intake [6]. Strengthened nutrition standards mandated under the Healthy, Hunger-Free Kids Act of 2010 (HHFKA) promote important improvements to school meal programs, such as increasing vegetable variety, offering only low- and non-fat milk, and establishing meal calorie minimums and maximums [7]. However, concerns about the amount of food selected but not consumed by students [8] threaten the viability of these standards [9,10]. Additionally, wasted food squanders the natural resources used to derive food and is a major contributor to climate change [11,12]. Traditional nutrition education interventions that target students’ selection, consumption, and waste of fruits and vegetables during school meals are rarely effective past the short-term [13,14], suggesting that a novel approach is warranted.

Emerging evidence suggests a link between young people’s interest in alternative food production practices, like locally grown foods and foods grown using sustainable agricultural techniques, and dietary quality [15,16]. Yet, the limited available evidence suggests adolescents may not be aware of the impact that their eating behaviors have on the environment [17]. Researchers in the United Kingdom [18], Canada [19], and Australia [20] have demonstrated the intersection between food systems and health in the public school setting, and in the United States, food systems education is considered a form of farm-to-school programs [21,22]. A recent systematic literature review of farm-to-school programs questioned the feasibility of incorporating these interventions into classroom curricula and identified the failure to quantify intervention fidelity as one of the major limitations of existing research on these programs [23]. Schools have educational priorities that may compete with health priorities [24], and the constrained budget, time, and staff of school systems [25,26] can make it difficult to sustain school-based health interventions in the short and long term. This makes schools an ideal setting for implementation science research, which examines the effective dissemination and implementation of evidence-based interventions in the real world, with a focus on evaluating program feasibility, acceptability, and fidelity [27].

Middle schools are an ideal setting for our student intervention since lessons on food systems concepts are well aligned with the academic learning standards required for middle school (grades 6–8) [28]. Also, compared to younger children, adolescent students are making more of their own food choices and may be better able to connect their food choice and waste actions to health and environmental consequences. Yet, there is little information on the impact of food systems education in this age group. The primary purpose of this study was to examine the impact of a student-driven sustainable food systems education and promotion intervention on adolescents’ food selection, consumption, and waste behaviors, particularly for fruits and vegetables, during school lunch. In addition, we aimed to understand the influence of the intervention on students’ knowledge and attitudes towards the food system and to estimate the intervention acceptability and fidelity.

## 2. Materials and Methods

### 2.1. Study Design and Setting

**Figure 1:**
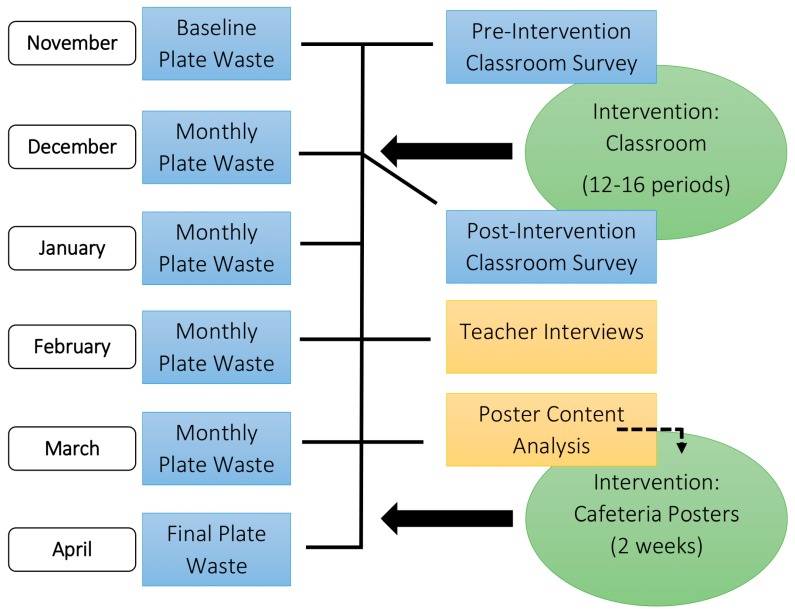
Overview of the Healthy Planet, Healthy Youth experimental embedded mixed methods design, including timeline of intervention and data collection. Rectangular elements illustrate data collection, where blue signifies quantitative data and yellow signifies qualitative data. Oval elements illustrate intervention points and duration of intervention. The dotted line indicates that the poster content analysis results were used to develop the cafeteria poster intervention.

The Healthy Planet, Healthy Youth (HPHY) study used an experimental embedded mixed methods design [29], in which the qualitative data were embedded within and generally played a supportive role in the non-randomized controlled trial which was primarily based on quantitative data. Figure 1 details the sequence of study events with qualitative data indicated by yellow boxes and quantitative date in blue boxes. In addition, a community-based participatory research approach was used [30], which promotes the value of community members as equal partners throughout the research process. The HPHY Advisory Committee met quarterly the year prior to the intervention and semi-annually during the intervention year. The HPHY Advisory Committee included school nutrition staff from three local school districts, staff from the state office of school nutrition, and university faculty with a variety of expertise, including science education, food safety, nutrition, and agricultural economics. HPHY was implemented in two Colorado middle schools within the same school district. The school nutrition programs at both middle schools had salad bars, scheduled lunch periods lasting 30–32 min, and offer vs. serve provisions which allowed students to decline some of the foods offered. The Blinded for Review Institutional Review Board approved this project.

### 2.2. Participant Selection and Recruitment

The local school district’s science education coordinator circulated recruiting advertisements to 6th grade science teachers via email. The district school nutrition program was actively involved in the HPHY Advisory Committee, facilitating engagement with the kitchen managers at the recruited middle schools. Participating teachers were provided a $250 cash incentive for their time, a copy of the curriculum, curriculum supplies, a one-hour in person training, and a curriculum outline that provided suggestions for how to amend the original curriculum so that it could be delivered in a shorter time frame.

All sixth-grade students enrolled in the science classes taught by the recruited teachers received the food system education intervention implemented as a unit during their science class. No parental consent, nor student assent, was required to receive the intervention, but written parental consent and electronic student assent were provided for all students participating in the classroom pre- and post-surveys. Verbal student assent was provided for all 6th–8th grade students participating in the monthly plate waste assessments and voting on food systems promotion posters; these two activities qualified for a waiver of parental consent.

### 2.3. Intervention and Theoretical Underpinnings

HPHY draws upon the Self-Determination Theory (SDT) [31], which underscores the importance of motivation quality, ranging from intrinsic to amotivation. In addition, SDT theorizes that people are more likely to achieve intrinsic motivation when their basic needs for autonomy, competence, and relatedness are met. In particular, the current study aimed to satisfy the participants’ need for relatedness by incorporating curriculum activities that facilitated student interactions, such as group projects and voting for food systems posters. Through these interactions we hypothesized that the intervention would nurture shared values of environmental conservation among students and would increase their motivation to make healthy food choices and waste less food.

An abbreviated version of an existing curriculum, Farm to Table and Beyond (FTTB) [28], was used for this intervention. This curriculum was selected since it is aligned to the required 6th grade science academic standards. The principal investigator and a local retired science teacher reviewed the entire curriculum and selected five lessons from FTTB that were central to the research questions, appeared feasible to implement, and provided maximum academic benefit to science teachers. These units included: Introduction to the Food System, Environmental Impacts of Food, Food Changes as it Moves through the Food System, Food Waste, and School Cafeteria Waste. Teachers were also encouraged to implement a supplementary lesson on Transporting Food. As a part of the School Cafeteria Waste lesson, students were tasked with estimating their personal lunch food waste over the course of one week. Teachers helped the students aggregate and graph the data as a part of a class project. For the culminating intervention project, students were asked to create a poster to teach the 7th–8th grade students in their school the most important thing they learned in the food systems unit.

For the promotion part of the intervention, researchers conducted a content analysis of the posters and used the findings to create professional-quality posters to promote waste reduction during school meals. At each school, the 6th–8th grade students voted on which poster they liked best using bingo chips, and the two posters with the most votes were hung in each school’s cafeteria during the final month of the intervention.

### 2.4. Measures and Data Collection

#### 2.4.1. Qualitative Measures and Data Collection

The qualitative data consisted of student posters and teacher interviews. Students at one school created individual posters, and the other created posters in groups; all were digitally photographed. Student names were removed or obstructed during photography. Teacher interviews were conducted using a structured interview protocol, audiotaped, and transcribed verbatim. Protocol questions included inquiries on overall feedback and adaptations used for each lesson, facilitators, and barriers to implementing the intervention, and the sustainability of the intervention. Transcriptions were assessed for quality. Written informed consent was acquired for each interview participant.

#### 2.4.2. Classroom Survey Measures and Data Collection

We adapted an existing 46-item, unpublished survey targeting 5th to 6th graders to evaluate FTTB [28]. The original FTTB survey focused on the nature and human relationship, student interest in science, and their attitudes towards healthy foods. We used a three-step process to ensure the validity and reliability of our adapted survey. First, we assessed survey constructs for our project by holding two 60-min focus groups (1 for boys, 1 for girls) with 8th grade students and revised survey items accordingly. Second, individual 30-min cognitive interviews were completed in July–August 2017 with rising middle school students at a school district summer program to establish face validity of the revised survey questions. The survey was updated to improve participant comprehension and promote increased congruence between researcher and participant understanding of key terms used in the survey [32,33]. Third, an online survey repeated twice within a 10–21 day timeframe assessed test-retest reliability. Participants (*n* = 65) were recruited through a direct mailing list of local families with children aged 11–13. Unreliable survey questions were not used in further analyses. Reliable survey questions were grouped according to pre-identified themes based upon the self-determination theory and curriculum units: relatedness, regulatory style, stewardship, food processing, local food, natural resources, packaging, food waste, climate change, food systems, and transport. Themes with at least two items showing acceptable internal consistency (Cronbach’s alpha greater than 0.7) were used in analysis. Final classroom survey scales included: relatedness (6 items), regulatory style (7 items), natural resources (2 items), and food packaging (3 items). Three food waste items that were reliable but did not fit into a scale score were also included.

#### 2.4.3. School Meal Component Selection, Consumption, and Waste Data Collection

Plate waste data collection was conducted one day per month for six months (November 2017–April 2018; Figure 1) at each school using a previously validated digital photography method [34,35,36]. Monthly plate waste assessments for each school typically occurred within seven calendar days of each other. Plate waste dates were chosen after consulting with the school principal to work around school events and field trips and according to the availability of research staff. Students had no advance knowledge of when data would be collected and were not told that the plate waste measures were related to the classroom curricular intervention. The baseline plate waste collection occurred prior to the start of the classroom lessons. Students went through the lunch serving line using the normal school procedures. Trained researchers met students at the cashier, obtained verbal assent, and completed tray tags to indicate the sex, grade, and selected food items for each student. Tray tags were pre-printed with the day’s menu options. Researchers circled the selected entrees, hot vegetables, whole fruit, beverages, and a la carte items on the tray tag, and documented selected salad bar items and the corresponding visual estimates of served portions. After students were finished eating lunch, their tray was brought to the research station located near the garbage cans. Researchers labeled trays with a unique tray number, measured beverage waste to the nearest 0.5 ounce using a liquid measuring cup, and then photographed the remaining food on the tray against a reference board with the camera 26 inches above at a 45-degree angle. Three to five reference foods of each item served that day were collected and photographed prior to the start of lunch. Reference foods were taken back to the lab and weighed to the nearest 0.5 g. An average weight for each reference food was calculated from the three to five reference food samples.

Photographs were independently, visually assessed for the percent of each food item wasted by two, trained researchers. A third researcher, experienced in the digital photography plate waste method, compared the two assessments. Estimates for percent wasted were confirmed identical or were averaged if the two estimates were within 20%. The third researcher reconciled any percent wasted estimates that differed more than 20%. Standardized weights and standardized percent wasted amounts were used when possible, such as items that could be broken down by food component (i.e., bread and bun estimates for sandwiches). Reference food weights were merged with portion/amount taken and percent wasted estimates to calculate the weight of each food item wasted.

#### 2.4.4. Research Staff Data Trainings

Researchers attended a 1.5-h data collector training which consisted of an overview of the study purpose and rationale, review of the data collection protocol and data collection sheets, hands-on practice assessing menu items selection and estimation of salad bar portion size selection, and expectations for professional conduct. The 1-h data analysis training consisted of an overview of the study purpose and rationale, orientation to reference photographs and standardized percent wasted amounts, and practice assessing the percent wasted of actual participant lunch trays.

### 2.5. Data Analyses

#### 2.5.1. Qualitative Analyses

Teacher interview transcripts were analyzed using ATLAS.ti (Version 8.0.4; Berlin, Germany, 2017). We used a single researcher, two-pass hybrid deductive–inductive qualitative approach, where the implementation science research questions informed the initial codebook and additional unique themes emerged during the coding process [37].

Digital photographs of student posters were also analyzed using ATLAS.ti (Version 8.0.4; Berlin, Germany, 2017). Seven food systems themes were identified a priori based upon the curriculum content and feedback interviews with classroom teachers: food waste prevention, food recovery, prevention of other related waste (packaging, food implements), recycling, reasons to reduce waste, natural resources, and growing your own food. A two-pass deductive content analysis coding method [38] was used by a single researcher to classify student messages written and drawn on the posters utilizing these a priori themes, and descriptive statistics were used to explore differences in theme frequencies across schools.

#### 2.5.2. Classroom Survey Pre and Post Data Analyses

Classroom survey analyses were completed using R 3.4.1 and the following packages: dplyr, ggplot2, lme4, lmerTest, and emmeans. Statistical significance was set at α = 0.05. The overall mean for each outcome measure was calculated for pre and post and compared using a paired t-test or paired Wilcoxon signed-rank test (for non-normal data). The mean difference between pre and post (post score − pre score) was calculated for each outcome measure by school and schools were compared using a Wilcoxon signed-rank test. Linear mixed models were used to assess change in outcome measures over time. All models included an individual random effect for unique subject ID to account for repeated measures (pre and post). Demographic factors (school, sex, race, ethnicity, how a student eats lunch, farm experience, garden experience, and cooking frequency) were included as fixed effects in all models to assess whether demographic groups differed in the outcome measure. For the demographic factors that had different pre to post trends, interaction terms were added individually to the mixed model to assess differences by demographic groups. Farm experience and garden experience were continuous variables calculated from a multiple answer question, where zero represented no experience and each additional experience (total of four for farming and three for gardening) added one. Cooking frequency was also a continuous variable from zero to seven and represented the average number of days per week a student helped make breakfast, lunch, dinner, and snacks. For each outcome measure, the model with the lowest Akaike information criterion was considered the final model for interpretation. Model assumptions were checked using the residuals versus fitted plots and Q-Q plots. Model results for fixed effects were investigated using a Type 3 ANOVA table, and estimated marginal means were used to investigate contrasts between time points and demographic factors.

#### 2.5.3. Plate Waste Data Analyses

Plate waste data analyses were completed using SPSS software (Version 24; Armonk, NY, USA, 2016). Statistical significance was set at α = 0.05. Descriptive statistics (overall means or frequencies for each outcome variable) were calculated for the intervention (6th graders) and control groups (7th–8th graders) at each of the six time points (pre-intervention through five month follow-up).

Food selection outcomes were binary (1 = student selected item from food group, 0 = student did not select item from food group) and were presented as the percent of participants who selected items from each food group (vegetables, fruit, entrée, and milk). Food consumption outcomes were continuous and expressed as the percent of each food group (vegetables, fruit, entrée, and milk) that each participant consumed. Food waste outcomes were continuous and expressed as the weight (in grams for solid food items, in fluid ounces for milk) of the food that was wasted or thrown away at the end of lunch. The vegetable selection and waste variables included both hot vegetables and vegetables from the salad bar, and the fruit selection and waste variables included both whole fruits and fruit from the salad bar. Logistic regression analyses controlling for participants’ gender and school were used to assess differences between the intervention and control group in food selection outcomes (vegetables, fruit, entrée, and milk) at key time points (pre-intervention, post-intervention, and five month follow-up). Two-way ANCOVAs were used to analyze the effect of condition (intervention vs. control group) and time point on food consumption and waste outcomes (vegetables, fruit, entrée, and milk). These analyses controlled for participants’ gender, the school that they attended, and the percent of entrée consumed. Estimated marginal means (adjusted to account for the influence of control variables) were used to compare outcomes between the intervention and control group. Post-hoc analyses (with Tukey corrections) were used to determine whether there were significant differences between the intervention and control group in consumption and waste at key time points (pre-intervention, post-intervention, and five month follow-up). These logistic regression and ANCOVA analyses were also repeated with school A and school B individually to investigate differences in outcomes by school.

## 3. Results

The HPHY education and promotion intervention was delivered to approximately 268 6th grade students between the two schools, and an additional 650 students in 7th–8th grade were exposed to the promotional food systems posters in the cafeteria (Table 1). There were four total 6th grade science teachers between the two schools, and all of them agreed to deliver the classroom intervention. The results section provides findings for the intervention fidelity and feasibility, poster content analyses, classroom survey, and plate waste outcomes.

### 3.1. Intervention Fidelity

Table 2 summarizes the student activities for each lesson. Few lessons were implemented as suggested in the curriculum outline. One universal adaption was incorporating suggested homework activities into classroom learning since neither school typically assigned science homework. For school A, one lesson was omitted and substituted with a video due to standardized testing-related changes to the daily school schedule that made it difficult to implement an interactive learning activity across all class periods. In School B, one class period was omitted due to overlap in the previous month’s science unit on climate change. In addition, the teacher at School B thought that her school was already progressively handling the food waste issue since they donate all cafeteria food scraps to a local pig farmer and omitted the Cafeteria Waste Inventory unit.

### 3.2. Teacher Feedback on Implementation: Feasibility, Acceptability, and Fidelity

Three of the four intervention teachers participated in individual interviews. Thematic analyses yielded six themes: age-appropriate content, student engagement, barriers and facilitators, teacher engagement, and building on related science topics. Definitions for each theme and example quotes are provided in Table 3. Taken together, the interview data underscore the importance of engaging teachers, as well as students. Teachers were overwhelmed with classroom time constraints and juggling the wide span of abilities among their students but reported that the freedom to tailor the curriculum to the needs of their students, amend lessons based on prior curricular topics, and adjust lessons due to school schedule changes were paramount to successfully implementing the intervention. In-person training, support from researchers, and an outline of strategies to amend the curriculum were also universally viewed as key facilitators.

Equally important, the teachers also agreed that adolescence was an ideal developmental period to teach food systems, particularly from a student-driven approach. One teacher said, “Honestly, [the best part of this unit was] the kids’ excitement and just their knowledge that they could impact something and that they had control over something at this age. I think they feel like so much of what they’re asked to do is from somebody else’s direction. So, the ability to be like, ‘I can make a choice in how much food I take’ appeals to them.” This view is supported by the high levels of reported student engagement in the topic. Yet, despite the developmental appropriateness of the topic, food systems was not an existing part of the science curriculum at either school. Both schools covered niche ecosystems in their ecology units, and School B had just finished a unit on climate change prior to starting the food systems education intervention. Teachers at both schools agreed that the intervention unit on food systems was an ideal complement to their existing science curriculum, particularly since it allowed them to cover the state-required scientific inquiry standards in a new way.

### 3.3. Content Analyses of Student Work and Student Voting Results

**Figure 2:**
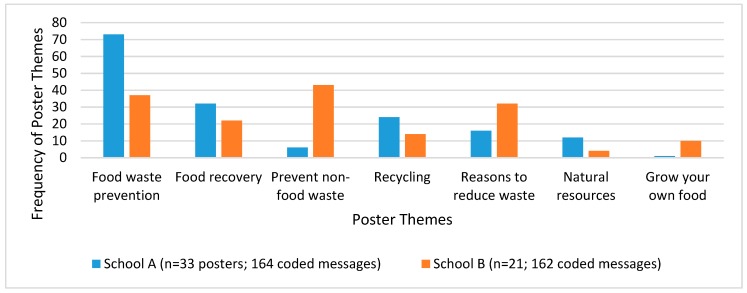
Student poster content analyses results, by school. A total of 54 posters were completed across both schools featuring 326 food systems themes. (Posters were completed in groups or individually, depending on teacher preference.) Food recovery are actions to avoid landfill disposal of wasted food, such as composting.

There were a total of 54 posters and 326 coded poster messages across the two schools. Figure 2 provides the frequency of the a priori poster message themes by school. Food waste prevention was the most common poster message overall, with school A featuring twice as many food waste prevention messages compared to school B. The most common poster message at school B was the prevention of packaging waste/disposable food implements, but this message was rarely included in posters at school A. Common stylistic factors of student posters included the incorporation of food into the letters of poster titles and statements to peers in the form of questions.

**Figure 3:**
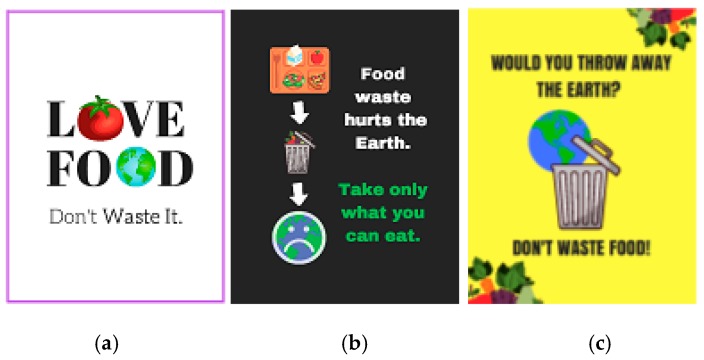
Food waste reduction poster winners, as voted on by school A (*n* = 347) and school B (*n* = 66) that were posted in school cafeteria during the final month of the intervention: (**a**) Top-voted poster at both schools (**b**) Second place poster at school A. (**c**) Second place poster at school B.

Figure 3 shows the posters receiving the most votes. These posters were displayed in the cafeterias during the final month of the intervention. Approximately 61.1% (*n* = 347) of the middle school students in School A and 55.1% (*n* = 66) in School B participated in the student poster voting.

### 3.4. Classroom Survey Results

A total of 268 students were enrolled in a 6th grade science class at the two schools and eligible for the study. About half of the students eligible (*n* = 130, 50.4%) had parental consent and 97 students completed the classroom survey (36.2% of eligible students, 74.6% of consented students). Due to a technical error with the online survey platform, some students were unable to finish their classroom survey. Table 1 provides the demographic characteristics of students participating in the classroom survey, and Table 4 provides outcome results from the classroom survey measures.

#### 3.4.1. Self-Determination Theory

**Figure 4:**
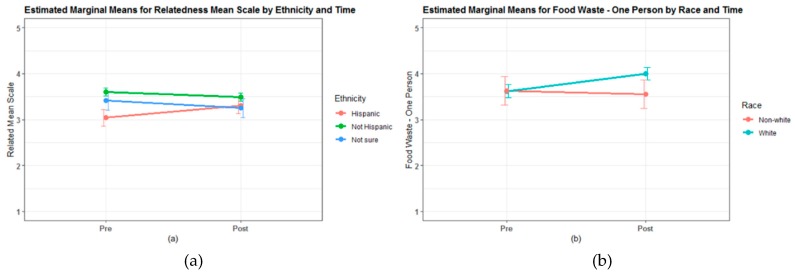
Interaction plots for (**a**) relatedness by time and ethnic group (*n* = 78; averaged over levels of school, sex, race, how a student eats lunch, farm experience, garden experience, and cooking frequency) and (**b**) food waste question (“I feel that one person’s food waste is bad for the environment”) by time and race group (*n* = 78; averaged over levels of school, sex, ethnicity, how a student eats lunch, farm experience, garden experience, and cooking frequency).

Overall, *relatedness* and *regulatory style* did not differ pre to post. However, change in *relatedness* was different depending on ethnicity group (time by ethnicity interaction: *F* = 5.33, *p*-value = 0.007) with Hispanic students significantly increasing in mean *relatedness* from 2.82 (standard error [*SE*] = 0.28) at pre to 3.21 (*SE* = 0.28) at post (Figure 4). Non-Hispanic students and those students who were not sure of their ethnicity did not differ in mean *relatedness* from pre to post. Change in *relatedness* did not differ among other available demographic characteristics and change in *regulatory style* was not different among any demographic groups.

#### 3.4.2. Food Systems and Food Waste Knowledge and Attitudes

Overall, only one of the food waste knowledge and attitudes measures was significantly different pre to post: “I feel that one person’s food waste is bad for the environment.” In addition to increasing overall, the change in response to this question differed by student race with white students increasing from 3.62 (*SE* = 0.14) at pre to 4.00 (*SE* = 0.14) at post (contrast *p*-value < 0.001; Figure 4). Students in non-white race categories were combined due to sample size and did not differ in response from pre to post. *Natural resource knowledge* was not significantly different pre to post overall or within each school group. However, while both schools had similar *natural resource knowledge* at pre, the two schools were significantly different at post (contrast *p*-value = 0.028). School A increased in *natural resource knowledge* from 3.51 (*SE* = 0.26) at pre to 3.71 (*SE* = 0.26) at post, where School B decreased from 3.33 (*SE* = 0.27) at pre to 3.18 (*SE* = 0.27) at post. *Food packaging knowledge* and the two remaining food waste knowledge and attitude measures did not change significantly pre to post overall or by any of the available demographic characteristics.

### 3.5. Plate Waste

**Figure 5:**
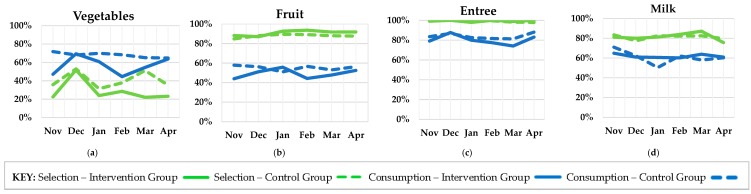
Selection (percent of students who selected food) and consumption (average percent of food consumed) outcomes at each of the six time points for the following food groups: (**a**) Vegetables (includes both hot vegetables and vegetables from the salad bar, (**b**) Fruit (includes both whole fruit and fruit from the salad bar), (**c**) Entrée, and (**d**) Milk. Sample size ranged from 85 to 112 for the intervention group and 145–187 for the control group across the six time points. Estimated marginal means are displayed for consumption variables while original frequencies are displayed for selection variables.

A total of 1596 plate waste observations occurred across the six data collection dates between the baseline (pre-intervention) time point and the five month follow-up. Frequencies for selection and estimated marginal means for consumption in the intervention and control groups at each of the six time points are provided in Figure 5. Analyses focused specifically on the differences between intervention and control groups at pre-intervention (*n* = 256), immediately post-intervention (*n* = 236), and the five month follow-up (*n* = 286). Across the six time points, participants were 43% female and 57% male. Approximately 37% of participants were in sixth grade (intervention group), 36% were in seventh grade (control group), and 27% were in eighth grade (control group).

Logistic regression results are provided in Table 5. At baseline, the odds of students selecting vegetables was significantly higher in the control group (35.9% selection) compared to the intervention group (22.5% selection). Immediately post-intervention, the intervention group increased their vegetable selection by 29.3 percentage points and differences between groups were no longer significant.

Two-way ANCOVA results for food consumption and food waste at baseline, post-intervention, and five month follow-up are provided in Table 6. While two-way interactions between condition and time point were not significant (across all six time points) for any food consumption or food waste variables, some differences between experimental groups were observed at specific time points. The estimated marginal means for the average consumption of vegetables was significantly lower in the intervention group (47.1%) compared to the control group (71.8%) at baseline. The estimated marginal means for the average consumption of fruit were significantly higher in the control group (57.9%) compared to the intervention group (44.0%) at baseline.

The estimated marginal means for the average hot vegetable waste were significantly higher in the intervention group (26.4 g) compared to the control group (6.1 g) at baseline. There were no significant differences in salad bar vegetable waste between conditions at pre- and post-intervention time points, but at five month follow-up, the control group (on average) wasted significantly more vegetables from the salad bar. There were no significant differences in salad bar fruit waste between conditions at pre- and post-intervention time points, but at five month follow-up the control group (on average) wasted significantly more fruit from the salad bar.

For School B, there were no significant differences in vegetable consumption between the control and intervention groups at baseline, the first post intervention assessment, nor the five month follow up assessment. On the other hand, School A had a significant difference in vegetable consumption at baseline between the intervention and control group, had no differences at the first post intervention assessment, and no differences at the five month follow-up assessment. (Stratified analyses by school not shown in tables or figures.)

## 4. Discussion

In this experimental embedded mixed methods study, adolescents who received a food systems education and promotion intervention increased their vegetable and fruit consumption relative to baseline, while the control group decreased their vegetable and fruit consumption. Although the percentage of intervention students selecting a vegetable was less than controls at the five-month follow-up, intervention students wasted less of the vegetables and fruit that they selected. These differences in wasted produce were primarily driven by reductions in salad bar fruit and vegetable waste, likely stemming from the autonomy students have in determining the portion size of salad bar items. Taken together, these findings suggest that food systems education can positively influence dietary behaviors among adolescent youth. However, the plate waste outcomes were not consistent over time with vegetable consumption rates approaching baseline levels three months after the intervention and then increasing back to the levels achieved at 1-month post intervention once the food systems cafeteria promotions were implemented. This may signify the waning influence of the classroom intervention over time, that was later bolstered by the food systems promotions at the end of the year. These changes over time suggest the need to find ways to reinforce the classroom curriculum messages throughout the school year.

The current study findings complement previous literature exploring the relationship between young people’s interests in sustainable food systems and diet quality and extends this connection to younger adolescents. Robinson-O’Brien et al. reported that people aged 15–23 years who valued at least 2 alternative agriculture practices, such as locally grown, non-processed, or organic foods, were more likely than their peers to have a dietary pattern consistent with the Healthy People 2010 objectives [15]. Similarly, Pelletier et al. concluded that young adults (mean age 21.9 years) who place a higher importance on alternative food practices consumed 1.3 more servings of fruits and vegetables, more dietary fiber, fewer added sugars and sugar sweetened beverages, and less fat relative to their peers who placed low importance on these practices [16]. The findings from the current study illustrates that a sustainable foods intervention can promote improved diet quality and reduced food waste among a younger population and used an objective dietary assessment method.

The present study findings contrast with the findings of Goldberg et al.’s study on a communications campaign intervention that focused on the overlap between healthy eating and eco-friendly behaviors aiming to improve the quality of foods brought from in packed lunches among 3rd and 4th graders [39]. The authors found no significant changes in the quality of lunch food brought from home and were unable to conclude that classrooms were an effective tool to facilitate changing the school meal related behaviors [39], whereas the present study was able to leverage classroom experiences in the school cafeteria. It is likely that the relationship between healthy eating and environmental sustainability was too complex to be effectively transmitted from classroom teachers to children to parents, and it is also possible that the adolescents in the present study may be a more developmentally appropriate audience to understand this complex relationship than elementary school students.

Traditionally adolescent behavioral nutrition interventions have utilized health or nutrition education to change dietary behavior, but have shown little effectiveness in this age group [40]. The current study used food systems education, using an approach informed by the self-determination theory [41], to influence adolescent behavior. The intervention provided opportunities for students to engage in conversations and activities with their peers to discuss issues related to planetary health, taught students that individual’s food decisions have important consequences, and reminded them that they have the power to change their own behaviors. These food systems concepts complement the underlying tenets of the self-determination theory: relatedness, competence, and autonomy. These concepts may also appeal to the increased concern for social justice that is often experienced during adolescence [42,43], further fostering intrinsic motivation to improve dietary behaviors. The teachers in the present study supported these constructs, universally viewing the food system lens as a developmentally appropriate strategy to influence adolescent eating and wasting habits. In addition, students successfully created a variety of relevant messages after being exposed to food systems education, also demonstrating their level of understanding of this important topic and that 6th grade is an appropriate age for food systems interventions.

The relationship between healthy eating habits and food waste reduction is complicated, as messages to reduce waste could potentially have unintended negative consequences, such as increasing portion sizes and ignoring satiety cues. In the present study, the measured food environment exclusively consisted of food served through the National School Lunch Program, which meet strict nutrition standards. Thus, increases in consumption of these foods may be viewed favorably. Additionally, selection and consumption were measured as a percentage and do not necessarily indicate that an increased amount of food was selected or consumed, particularly since portions of salad bar items were self-selected. In the present study, significantly more control students a vegetable, while intervention students wasted significantly less vegetables and fruit from the salad bar at the five-month follow-up. These findings suggest that the differences in dietary behaviors are unlikely to be explained by overeating behaviors and are more likely attributed to intervention students being more selective about what and how much food they put on their trays. Taken together, this study underscores the importance of reporting the amount of food wasted, not just selection or consumption percentages.

The feasibility of classroom-based farm-to-school programs has previously been questioned [23]. Yet, the teachers in the current study reported that the intervention was feasible and acceptable, as evidenced by their continuation of the intervention without researcher involvement the following year. Teachers viewed the freedom to adapt the lesson to their classroom as essential to feasibility and acceptability. However, these adaptations resulted in important differences in how the curriculum was implemented between schools, and these differences impacted student intervention experiences. The school-level differences in poster themes across schools and differences in the natural resources’ knowledge scores mirror the variation in intervention implementation. The lack of significant differences in vegetable consumption between the intervention and control groups at School B also suggest that the desirable changes in vegetable consumption and waste for the overall sample were primarily driven by School A, which also corresponds to the implementation differences on the cafeteria waste unit. These findings demonstrate the importance of incorporating intervention fidelity measures and other implementation metrics in school-based nutrition research.

This study also has important limitations to consider. First, the plate waste data uses controls that are 1–2 years older than the intervention participants. This age difference is consequential due to the likelihood of the older students experiencing increased growth velocity that occurs during puberty. Subsequently, we believe our consumption and waste estimates are conservative given that older children are likely to eat more and waste less relative to younger children. Second, we were unable to conduct plate waste on the same menu days throughout the year. While this does not impact comparisons between comparison and control groups, it makes it difficult to assess trends over time. Third, our implementation assessment only included qualitative measures. Quantitative measures would have allowed us to compare implementation indices relative to other published literature. Fourth, some of the classroom survey scales had poor internal consistency and were not included in our analyses. This may be a consequence of the interrelated nature of food systems concepts, making it difficult to differentiate sub-sections from one another. Fifth, the classroom survey, in particular, had low sample size. This limitation may have contributed to the inconsistencies in the survey findings, such as only 1 of the 3 food waste questions showing significant change. Finally, we did not include any academic outcome measures. Little is known about how nutrition and food systems education impact academic outcomes [44,45]. In order for more schools to incorporate these topics into their curricula, more evidence is needed to link to educational outcomes [23,45]. The teachers in our study were motivated by the high quality writing and group activity assignments submitted by their students during the intervention; this perceived impact on student academic performance likely influenced the teachers’ desire to continue the program without researcher support. Quantitative evidence on the impact to academic outcomes may facilitate widespread adoption of a food systems curriculum in middle schools.

## 5. Conclusions

Adolescence is an ideal developmental period for food systems education. Our study demonstrated that food systems education implemented by science teachers can be used to improve fruit and vegetable consumption and wasting behaviors during school meals. Further, teacher freedom to adapt the lessons was viewed as essential for intervention feasibility and acceptability, but the adaptations hindered implementation fidelity. Additional research is needed to understand the impact of food systems education, particularly food waste reduction messages, on food served outside of the National School Lunch Program, such as desserts and other energy-dense foods. Future studies should also examine academic outcomes to aid widespread incorporation of food systems concepts into school curricula and investigate strategies to reinforce food systems concepts after the classroom instructions end to promote long-term dietary change.

## Figures and Tables

**Table 1:** Demographic characteristics of intervention schools (*n* = 2) and sample demographics of students participating in the classroom survey.

	School A	School B
**School Enrollment ^1^**		
**Total**	568	129
**Sex, *n* (%)**		
Male	297 (52%)	64 (50%)
Female	271 (48%)	65 (50%)
**Race, *n* (%) ^2^**		
White	254 (45%)	106 (82%)
Hispanic	274 (48%)	10 (8%)
Non-White or Non-Hispanic	40 (7%)	13 (10%)
**Classroom Survey Sample**		
**Total**	56	41
**Sex, *n* (%)**		
Male	23 (41%)	20 (49%)
Female	31 (55%)	19 (46%)
Not reported	2 (4%)	2 (5%)
**Age, mean (*SD*)**	11.31 (0.47)	11.32 (0.52)
**Race, *n* (%) ^3^**		
White	40 (71%)	32 (78%)
Non-White	6 (11%)	3 (7%)
Unsure or not reported	10 (18%)	6 (15%)
**Ethnicity, *n* (%)**		
Hispanic/Latino	12 (21%)	5 (12%)
Not Hispanic/Latino	34 (61%)	29 (71%)
Not sure or not reported	10 (18%)	7 (17%)
**How students eat lunch on school days, n (%)**		
School lunch	25 (45%)	14 (34%)
Bring lunch from home	11 (20%)	15 (37%)
Combination of school lunch and food from home	11 (20%)	6 (15%)
Choose not to eat lunch	4 (7%)	5 (12%)
Not reported	5 (9%)	1 (2%)
**Farm Experience ^4^, *n* (%)**		
Live on a farm	2 (4%)	3 (7%)
Worked on a farm before	14 (25%)	10 (24%)
Family member works on a farm	8 (14%)	6 (15%)
Visited a farm before	29 (52%)	23 (56%)
No farm experience	4 (7%)	4 (10%)
Not reported	14 (25%)	3 (7%)
**Garden Experience ^4^, *n* (%)**		
Garden at home	23 (41%)	23 (56%)
Help with school/community garden	6 (11%)	4 (10%)
Gardened in the past	19 (34%)	15 (37%)
Do not garden	2 (4%)	5 (12%)
Not reported	14 (25%)	3 (7%)
**Cooking frequency ^5^ (overall), mean (*SD*)**	3.85 (1.84)	3.35 (1.91)
Breakfast	4.17 (2.48)	3.02 (2.67)
Lunch	3.00 (2.27)	2.81 (2.20)
Dinner	3.41 (2.57)	3.08 (2.44)
Snacks	4.66 (2.48)	4.43 (2.65)

Notes: *SD*: standard deviation; ^1^ School enrollment is for grades 6 to 8 only and sourced from administrative data. ^2^ Non-White or Non-Hispanic races include Black, Asian, American Indian or Alaska Native, and 2+ Races; ^3^ Non-White races include Black, Asian, American Indian or Alaska Native, Native Hawaiian or Other Pacific Islander, 2+ Races, and Other; ^4^ Student could choose multiple responses, so percentages do not sum to 100%; ^5^ Cooking frequency was reported as average days per week each student helped prepare the specified meal; results are average days per week; overall is the average of all meal categories.

**Table 2:** Intervention implementation summary by school.

	Summary of Class Activities Implemented	
Curriculum Unit: Aim	School A	School B
Introduction to the Food System: To assess what we already know about the food system and how it affects the environment	Students selected a food item of their choice and drew a diagram of all the steps that the food goes through from farm to table.	Students drew diagrams of the steps that apples and applesauce go through from farm to table.
Transporting Food: To gain an understanding of the role transportation systems play in food systems. (This was an optional supplementary lesson.)	Lesson omitted.	Students were each assigned information summaries to review about one of the following: food transport via airplane, railroads, inland waterways, ocean freighter, or truck. Later, students were put into groups with students who had been assigned different transport options from themselves. Each student had to teach their group about the advantages and disadvantages of their assigned method of food transport.
Environmental Impacts: To construct knowledge about the importance and use of natural resources, including fossil fuels	Students did a Point of View activity where they were each assigned different roles to play at a town meeting, such as Soil Scientist or Food-Packaging Manufacturer. The purpose of the town meeting was to decide whether or not to allow people to cut down unlimited trees on the nearby mountain to use in manufacturing or to place strict limits on the number of trees that can be cut down. Students had to build their argument, write their argument, review other students’ written arguments, and vote to determine the outcome.	Lesson omitted.
Food Changes as it Moves through the Food System: To construct knowledge about the environmental effects of food processing	The lesson was replaced with a video about the impact of human consumption of food, everyday products, and fuel on the planet.	Students reviewed how trends in food packaging and garbage disposal have changed over time. Students were asked to create their own snack company that is profitable, yet minimizes the impact on the environment. Students mapped out the farm to table process of all ingredients in their company’s food product, including food and packaging waste and fuel sources used to power their company’s factories.
Food Waste: To analyze the amount of waste individuals generate and to develop a method for surveying school-cafeteria waste	The lesson was omitted, and students were assigned to read and answer questions on a magazine article on food waste. They were also challenged to track their weekend food waste at home. Findings were aggregated by the teachers and reviewed with students.	Lesson replaced with teacher- facilitated discussion on single-use products vs. reusable products, with an emphasis on cups and silverware.
Cafeteria Waste Inventory: To collect, analyze and utilize data about food-related waste in the school cafeteria.	Students were given an index card to document how much food they threw away at lunch and why over the course of one week. They graphed the results as a class.	Lesson omitted
Share Most Important Message: To share the most important thing they learned in the unit with other students	Students created posters to share their messages, either individually or in groups.	Students created posters, individually, to share their messages.
Total classroom days devoted to the unit:	16 days	12 days

**Table 3:** Middle School Teacher Interview Theme Results (*n* = 3).

Theme	Illustrative Quote(s)
Adolescent development	I would suggest for other middle school students to use [the curriculum]. I think it’s an appropriate time [for it]. [Middle school students] have enough of a world perspective to know that there is stuff outside the grocery store and outside their own kitchen. But I think we kind of take for granted that people notice things and unless we teach it, they won’t [notice] because it’s not part of everyday experience. I think [this topic] is perfectly appropriate for middle school.
	I think it’s really relevant because [my students should] be more aware of the world around them, to be aware of some of the hardships that their families face. So, it’s like, “Oh! That did cost mom and dad money when they threw this thing away.” So, I think it’s a very timely thing for them to be aware of.
	I think [this topic] is valuable. Sixth, seventh and eighth grade is when they start making food choices of their own. They might be like out with friends and buy a soda. So, I think the packaging piece was valuable for them to think like, okay, where this is going to go once it leaves me?
Student engagement with the material and each other	This was probably the best quality work of the two posters we had to make and the writing piece [from this unit]. That’s the best quality work I’ve seen almost all semester. Because it was meaningful to them, it was impacting them.
	I think it was cool for them to be like, “Hey, that’s my index card, that’s my data, this isn’t just some story problem out of a textbook. This is like my lunch last week.” I think that was good, and they took some ownership of [the cafeteria waste assignment] that way.
	I think just because it was more engaging, they were more willing to take a risk and work with somebody that they hadn’t worked with before. So, that’s not content specific, but I think it speaks to the content and how engaging it is because it was cool to see kids specifically like between ethnic groups [work together].
Barrier: Time constraints	Unfortunately, the thing I would do differently would be I would back away, from winter break a tiny bit. Just because I think we could have a more meaningful discussion about, ‘did you have change over the week,’ ‘did you have your mom pack your lunch differently’ or ‘did you ask for different things when you went through the line.’ I would love to have had like an extra day, to have done some sort of post discussion or debriefing or survey or something like that, it just was like we never had time to do that.
	Yeah, I liked it a lot I guess I would like more time with it. I think I did it in 2 weeks or 3 weeks and it still felt like I needed more time.
Barrier: Wide span of student reading-levels and abilities	I have some sixth graders that read on like a fourth-fifth grade level. Some sixth graders read on like an eighth-grade, ninth grade level. So, it might be cool if [in the future] there were reading exerts that were tailored to that.
	We have a huge span of students’ experiences, capabilities and language capabilities… I think we have some students who probably could have developed their own research questions and probably could have made their own graph without any, or very little support. I have some students who, just a handful, but probably never understood why we were asking them to keep track [of their food waste]. So, a huge range. To try to close that gap [teacher name] and I [assigned the questions of] how many things are you throwing away and why [for each] day [during school lunch]. We felt like that was something that was approachable for almost all of our students, that they could do quickly, and that they wouldn’t blow off so that they didn’t lose their basketball time [at recess].
Facilitator: Academic standards	[Any new curriculum must] support stuff we are already doing because we don’t have time as teachers, or days in the classroom to add in something that’s totally new that we have never tried before that we don’t know if it’s going to work or support our curriculum. If it supports our curriculum and it’s a fresher more engaging way to do it, then absolutely, but if it’s going to be a ton of work and we are not sure if it’s going to support what we are doing I would say, I would be reluctant to do it.
	So, we used this as a culminating piece of our ecology unit. So, we’d already been discussing like populations, niche ecosystems that kind of thing. So, it was taking the application of what they’d already learned about an ecosystem and kind of giving it a real-world application for them.
	The lessons are good. I think [the curriculum] takes things that we have to teach as [required academic] standards but puts them in an application that is often overlooked.
Facilitator: Curriculum outline	I think if you just gave a teacher this textbook, they’d never open it, as beautiful as it is. I think once a week somebody gives me a book and is like, “Oh, it’s great, just read it. I just found this. I had it 10 years ago; it’s all about this new thing about teaching Math.” I’m like great, you love your resources, but [teaching resources] need to be accessible and that’s what this [points to curriculum outline] is like. [The curriculum outline] made [the curriculum] accessible.
	I feel like you guys picked the lessons that were feasible, if not easy, to connect to one another. So, that is good, that’s a huge amount of work just to go through that book and pull out meaningful lessons because we can’t do them all.
Facilitator: support from researchers	I really appreciated the meeting with [Researcher Name] at the beginning with all of the teachers. We could all give our feedback and just how open she was with, “Call me if you have any questions, here is my e-mail and we can send a grad student.” I felt very well supported by her. I appreciated the outline. I appreciated the textbook… This is way more than I’ve ever gotten from anybody else. Like I said, a lot of people would be like, “Here’s a unit that you can do but you have to find the resources. Here are the resources, but how do you structure it and sequence it?” and I got both. It was like a gift.
	You guys did a great job, you were available and prompt, but you weren’t like staring over our shoulder or second-guessing the choices we made. It was great.
	That was awesome- you coming in and sitting down and going over [the curriculum outline]. Because you weren’t going to give somebody a [curriculum] book like this and they were going to be like, “Yeah, no, I’m not doing this.” So, you coming in and making it like, “Okay, here’s the bare bones of what we want you to do,” and just like running us through it really quick. So, then we could sit down as a team and go, okay, how do we modify this so it fits the needs for our students and they get something out of it?
Facilitator: teacher freedom to adapt the curriculum	Yeah, I don’t want somebody to tell me how to teach it because they might be a great teacher, but they don’t know my students the way I do.
	I really appreciated how open [Researcher Name] was to [us adapting the curriculum]. That made it really easy as a teacher because I think if she hadn’t said that and she had said ‘follow this piece by piece,’ I would’ve been overwhelmed and not done it. Because you have to adapt it for what your kids already know. This kid needs an extension; this kid needs support. So, if I were unable to make changes to it like I did, I don’t think I would have done it. So, that helped a lot.

**Table 4:** Comparison of changes in classroom survey measures pre to post.

	Overall Mean (*SD*)		*p*-Value ^1^	Mean Difference (*SD*) Pre to Post ^2^		*p*-Value ^3^
	Pre	Post		School A	School B	
**Relatedness ^4^**	3.44 (0.78)	3.39 (0.73)	0.703	−0.10 (0.65)	0.04 (0.51)	0.258
**Regulatory style ^5^**	2.94 (0.82)	3.00 (0.84)	0.656	−0.01 (0.78)	0.18 (0.77)	0.079
**Natural resource scale** ^4^	3.26 (0.92)	3.31 (0.98)	0.784	0.17 (0.89)	−0.18 (0.80)	0.037
**Food packaging scale** ^4^	2.37 (1.09)	2.47 (1.06)	0.515	0.11 (0.83)	0.15 (0.72)	0.976
**Food waste:** *I try to limit how much food I throw away* ^4^	3.56 (1.07)	3.69 (1.04)	0.321	0.20 (1.04)	0.13 (1.07)	0.974
**Food waste:** *When I am eating, I think about where my food came from* ^4^	2.56 (1.19)	2.64 (1.11)	0.603	0.11 (1.25)	0.08 (1.01)	0.702
**Food waste:** *I feel that one person’s food waste is bad for the environment* ^4^	3.54 (0.86)	3.80 (0.78)	0.044	0.26 (0.98)	0.25 (0.77)	0.833

Notes: *SD*: standard deviation; ^1^ Paired t-test (for normal data) or paired Wilcoxon signed-rank test (for non-normal data); H_0_: mean difference = 0; ^2^ Mean difference: Post score-Pre score; ^3^ Wilcoxon signed-rank test; H_0_: mean difference = 0; ^4^ Responses were a 5-point Likert scale; ^5^ Mean from seven questions with the same five response categories that classified respondents into a spectrum of regulatory style: amotivation (1), external motivation (2), introjected motivation (3), identified motivation (4), intrinsic motivation (5).

**Table 5:** Influence of Condition (Intervention vs. Control Group) on Food Selection at Pre-Intervention, Post-Intervention, and Five Month Follow-Up.

	Pre-Intervention (*n* = 256)			Post-Intervention (*n* = 236)			Five Month Follow-Up (*n* = 286)		
	Intervention % Selected	Control % Selected	*p*	Intervention % Selected	Control % Selected	*p*	Intervention % Selected	Control % Selected	*p*
**Food Selection (% of students)**									
Vegetables	22.5%	35.9%	0.021	51.8%	53.0%	0.880	23.2%	35.3%	0.054
Fruit	88.2%	84.8%	0.500	87.1%	88.1%	0.750	91.9%	87.7%	0.380
Entrée	99.1%	99.3%	0.875	100%	100%	-	100%	97.9%	0.996
Milk	81.1%	83.4%	0.438	80.0%	77.5%	0.859	75.7%	79.7%	0.121

Note: The *p*-values presented are for the influence of condition (intervention vs. control) on food selection outcomes at each time point.

**Table 6:** Differences in Food Consumption and Waste between Intervention and Control Groups at Pre-Intervention, Post-Intervention, and Five Month Follow-Up.

	Pre-Intervention			Post-Intervention			Five Month Follow-Up		
	Intervention Mean	Control Mean	*p*	Intervention Mean	Control Mean	*p*	Intervention Mean	Control Mean	*p*
**Food Consumption (% consumed)**									
Vegetables	47.1%	71.8%	0.006	69.4%	68.1%	0.848	63.8%	64.8%	0.905
Fruit	44.0%	57.9%	0.009	51.1%	56.3%	0.365	52.5%	56.4%	0.454
Entrée *	79.1%	83.5%	0.207	87.7%	86.8%	0.821	83.1%	88.1%	0.143
Milk	64.9%	71.0%	0.265	61.0%	62.4%	0.815	61.0%	60.3%	0.891
**Food Waste (grams wasted)**									
Hot Vegetable	26.4	6.1	0.015	15.4	19.2	0.384	9.5	9.8	0.965
Salad Bar Vegetable	26.6	19.0	0.466	15.1	18.5	0.756	24.2	50.1	0.029
Whole Fruit	79.9	68.9	0.241	61.2	52.4	0.384	62.0	67.1	0.577
Salad Bar Fruit	51.6	55.7	0.737	49.1	69.9	0.110	46.1	70.8	0.036
Entrée *	38.8	28.4	0.088	19.6	20.6	0.876	33.8	25.0	0.142
A La Carte	0.8	0.2	0.648	2.2	0.2	0.091	0.0	0.1	0.918
Milk †	2.9	2.3	0.219	3.3	3.1	0.782	3.2	3.2	0.940

Note. Estimated marginal means (controlling for gender, school, and entrée consumption) are displayed. The p-values presented are for differences between conditions (intervention vs. control) on food consumption and waste outcomes at each time point. The mean difference is significant at α = 0.05 for H0: The population mean difference is zero. Consumption means for vegetables and fruit include vegetables and fruit from the salad bar. Hot vegetable, salad bar vegetable, whole fruit, and salad bar fruit food waste outcomes are mutually exclusive. Consumption (*n* = 76–250) and waste (*n* = 52–245) depending upon time point and reimbursable meal component. * Entrée analyses did not control for entrée consumption. Entrées include combined protein and grain meal components. † Milk waste is displayed in fluid ounces.
